# Molecular Insights into Cardiovascular Disease: Unraveling Pathways for Diagnosis and Treatment

**DOI:** 10.3390/ijms26052067

**Published:** 2025-02-27

**Authors:** Dai Yamanouchi

**Affiliations:** 1 Department of Vascular Surgery, Fujita Health University, Toyoake 470-1192, Aichi, Japan; dai.yamanouchi@fujita-hu.ac.jp or yamano@surgery.wisc.edu; 2 Division of Vascular Surgery, Department of Surgery, University of Wisconsin School of Medicine and Public Health, Madison, WI 53705, USA

## 1. Introduction and Significance

Cardiovascular diseases remain a leading cause of morbidity and mortality worldwide, with aneurysmal diseases standing out as particularly insidious threats [1]. Aneurysms—characterized by the localized dilation and weakening of blood vessel walls—pose a significant health risk due to their potential for rupture and the ensuing life-threatening hemorrhage. In particular, abdominal aortic aneurysm (AAA) often progresses silently and is frequently underdiagnosed, yet is burdened with an alarmingly high mortality rate (estimated 65–85%) upon rupture [2,3]. These statistics underscore the urgent need for deeper insights into aneurysm pathogenesis and for improved strategies to detect, prevent, and treat this condition before catastrophic events occur. Understanding the molecular pathology of aneurysms is crucial, as it can reveal the fundamental mechanisms driving vessel wall degeneration and identify points of intervention to halt disease progression [4].

In recent years, growing research efforts have focused on unraveling the complex molecular underpinnings of aneurysm formation and progression in the broader context of cardiovascular [5]. This Special Issue of the International Journal of Molecular Sciences was conceived to highlight these advances and stimulate further discovery in the field. Key topics of interest include vascular remodeling, extracellular matrix (ECM) degradation, the role of inflammatory signaling pathways, and the identification of novel molecular targets for therapy [6]. By exploring these themes, researchers aim to paint a more complete picture of aneurysm pathobiology—from the cellular and molecular factors that weaken the vessel wall to innovative interventions that could fortify it. The collection assembled in this issue spans a diverse array of studies, reflecting the multifaceted nature of cardiovascular pathology. In the following, we provide an overview of the key themes and contributions, emphasizing their findings, potential impact, and translational significance for diagnostics and therapeutics in cardiovascular disease.

## 2. Key Themes in Molecular Mechanisms, Pathogenesis, and Novel Therapeutic Strategies in Cardiovascular Disease

Vascular Remodeling and Matrix Degradation: Aneurysm development is fundamentally a problem of altered vascular structure—the vessel wall undergoes remodeling that includes ECM breakdown, smooth muscle cell loss, and architectural weakening. Several contributions in this issue shed light on these processes [7,8]. For instance, the role of microbial factors in AAA pathology is explored by Nemes-Nikodém et al. [2]. Their study compares the microbiome of aneurysmal aortic walls (and intraluminal thrombus) to gut, blood, and healthy vessel controls in AAA patients. They discovered that certain bacterial genera—including Acinetobacter, Burkholderia, Escherichia, and Sphingobium—are significantly more abundant in aneurysm tissue than in normal vessel walls [9]. Notably, while some of these bacteria likely translocate from the gut, others appear to originate from environmental sources (such as the oral cavity or skin), insinuating multiple routes by which microbes may colonize the aneurysm site. This colonization is not merely a benign presence; the authors identified bacterial enzymatic activities with direct pathological relevance. Enzymes such as peptidylprolyl isomerase, found enriched in the aneurysm-associated microbiome, can have aggravating effects on aneurysm formation by contributing to ECM degradation or altering cellular. In contrast, other microbial enzymes (e.g., certain ATPases) present in healthy vessel walls may exert protective effects, suggesting a complex interplay where dysbiosis might tip the balance toward matrix destruction. These findings highlight an emerging paradigm: that vascular remodeling in aneurysms could be influenced not only by the host’s own cells and enzymes (such as matrix metalloproteinases) but also by microbiota [10,11,12,13]. This novel insight opens the door to considering microbial composition as both a biomarker and a potential therapeutic target (for example, through modulation of the gut microbiome or targeted antimicrobial strategies) in the context of aneurysm management.

Genetic factors also play a critical role in predisposing to or modulating vascular remodeling and aneurysm risk. In this issue, Goncharova et al. investigated the genetic underpinnings of thoracic aortic aneurysm (TAA) in a cohort of Russian patients with nonsyndromic sporadic [14]. Using massive parallel sequencing of 53 genes known to be associated with hereditary aortic disease, they sought variants that might explain aneurysm susceptibility. Interestingly, the study did not find any definitively pathogenic mutations in these patients; however, it identified a number of variants of uncertain significance (VUS) in a subset (~9.8%) of individuals. Notably, some of these VUS were located in genes with known roles in aortic disease, such as FBN1, COL3A1, and MYH11—genes definitively linked to connective tissue integrity and aneurysm syndromes. Others were in genes with more tentative or emerging associations (NOTCH1, COL4A5, PLOD3). The presence of these VUS suggests that even in apparently “sporadic” cases, underlying genetic variants may contribute to aneurysm pathogenesis or interact with other risk factors. The authors emphasize that further functional studies and familial screenings are needed to determine whether these variants incline patients toward disease (perhaps as low-penetrance risk alleles). This contribution underscores the complexity of the genetic landscape in vascular diseases—beyond clearly deleterious mutations, subtler genomic variants may influence the resilience of the vessel wall. In a translational sense, such work calls for improved genomic diagnostics and variant interpretation in cardiovascular medicine: as our understanding of these uncertain variants grows, they could be reclassified and eventually inform personalized risk assessment, early screening of at-risk relatives, or even genotype-tailored therapies in aneurysm patients.

Inflammatory Signaling Pathways and Cellular Mechanisms: Inflammation is a well-recognized driving force in aneurysm expansion and rupture. Chronic infiltration of macrophages and lymphocytes into the vessel wall leads to the release of proteases, cytokines, and reactive oxygen species that damage the matrix and weaken the vessel. Two studies in this special issue delve into the molecular regulation of inflammation in the context of aneurysmal and arterial disease, offering insight into potential molecular targets for therapy. Elbialy et al. examine the relationship between autophagy (the cellular self-cleanup process) in macrophages and inflammatory signaling [15]. Autophagy is known to regulate inflammation by removing damaged organelles and inflammasome activators; when autophagy is impaired, an exaggerated inflammatory response can result. The authors used an in vitro macrophage model (THP-1 cells) to simulate autophagy deficiency and observed a pronounced upregulation of NF-κB-mediated inflammatory genes under these conditions. Intriguingly, we discovered an alternative pathway for macrophage activation: in autophagy-impaired macrophages, inflammation was not solely driven by the known inflammasome–IL-1β axis, but also by activation of the ATM (ataxia telangiectasia mutated) kinase pathway leading to NF-κB activation. Pharmacologically, they showed that this effect could be rescued by treatments that mitigate cellular stress—either broad-spectrum antioxidants or a specific ATM inhibitor (AZD0156) effectively blunted the inflammatory gene expression surge. Furthermore, the study contrasted classically activated M1 macrophages (pro-inflammatory) with alternatively activated M2 macrophages (anti-inflammatory) in the context of autophagy. We found that M1 polarization inherently suppresses autophagy and induces DNA damage (potentially triggering ATM-mediated inflammation), whereas M2 polarization promotes autophagy and thereby reduces DNA damage and inflammation. Encouragingly, enforcing autophagy activity or inhibiting ATM during M1 macrophage activation shifted cells toward a less inflammatory state, highlighting the autophagy–ATM axis as a crucial moderator of macrophage-driven inflammation. This work identifies a promising molecular target—the ATM pathway—for therapeutic intervention to reduce vascular inflammation. In diseases like AAA and atherosclerosis, where macrophage-driven chronic inflammation contributes to tissue destruction, strategies to boost autophagy or selectively inhibit pro-inflammatory signaling (e.g., ATM or downstream NF-κB) could emerge as viable therapies. The implication is distinctly translational: existing ATM inhibitors (developed in oncology) or antioxidant compounds might be repurposed to dampen vascular inflammation and slow aneurysm growth, a hypothesis warranting further in vivo investigation.

Complementing this cellular-level perspective, Zalewski et al. provide a broader look at inflammatory signaling by profiling key regulators of angiogenesis and inflammation in patients with AAA [16]. Their study measured the expression of multiple genes (such as ANGPT1, CXCL8 (IL-8), PDGFA, TGFB1, VEGFB, VEGFC) in peripheral blood mononuclear cells, as well as circulating protein levels of growth factors like TGF-α, TGF-β1, and VEGF-A/C in individuals with AAA versus controls. The results revealed significant dysregulation of many of these factors in AAA patients: for example, pro-angiogenic and pro-inflammatory mediators were found at altered levels compared to individuals without aneurysms. Notably, factors like VEGF and TGF-β—which have context-dependent roles in vessel wall stability and inflammation—were significantly different in the AAA group, suggesting a disturbance in the balance of signaling required to maintain vascular integrity. While none of the measured factors correlated with aneurysm size, their abnormal levels in patients highlight that even early or small aneurysms are accompanied by systemic molecular. These dysregulated molecules present attractive candidates for biomarkers and potential therapeutic targets. If specific cytokines or growth factors are consistently elevated or depressed in AAA, they could be developed into blood tests to improve early detection of aneurysm or to monitor disease progression. Likewise, therapies that modulate these pathways (for instance, anti-inflammatory biologics or angiogenesis inhibitors/stimulators) might be explored to correct the pathogenic signaling milieu. The authors indeed suggest that the identified factors provide new insight into molecular pathways of AAA development and could serve as diagnostic or therapeutic targets moving forward. In summary, by mapping the inflammatory and angiogenic signature of AAA, this study adds to our understanding of how immune and growth-factor signaling drives aneurysm pathogenesis and lays groundwork for translational research on biomarkers and targeted interventions.

Innovative Diagnostics and Therapeutic Strategies: A prominent goal of molecular research in cardiovascular disease is to translate discoveries into better diagnostic tools and treatments. This special issue features noteworthy examples of such translational potential, from advanced imaging techniques to novel pharmacological interventions. On the diagnostics front, Hof et al. demonstrate the power of high-resolution vascular ultrasound for in vivo assessment of arterial pathology in mouse models of atherosclerosis and AAA [17]. Noninvasive imaging approaches are invaluable for early detection and longitudinal monitoring of disease, and this study provides an elegant validation of ultrasound as a research and potentially clinical tool for aneurysm surveillance. Using ApoE-deficient mice (prone to atherosclerosis) and Angiotensin II-induced AAA models, the authors showed that ultrasound-based measurements could sensitively detect changes in the arterial wall long before catastrophic events [18]. They observed, for instance, that global radial strain—an index of arterial wall elasticity—was markedly reduced in animals developing AAA (dropping from ~24% in healthy state to ~12% in aneurysmal aortas) and similarly attenuated in atherosclerotic carotid arteries. In aneurysm-bearing mice, vessel distensibility declined, reflecting stiffening, while in atherosclerotic mice the intima–media thickness increased, reflecting plaque buildup. These imaging findings were corroborated by histological analysis, which revealed increased collagen deposition and medial area in diseased vessels. Importantly, ultrasound-derived metrics strongly correlated with histopathological measures of damage, highlighting that imaging can serve as a reliable surrogate for tissue changes. The conclusion from Hof et al. was that high-resolution ultrasound can effectively trace early alterations in arterial wall properties in living animals, providing a window into disease progression that is both noninvasive and real-time. From a translational perspective, this work underscores how advancements in imaging technology might improve human health: similar ultrasound techniques could be applied in clinical settings to screen individuals at risk (e.g., older patients or those with genetic predisposition to aneurysm) and to monitor aneurysm growth or regression in response to therapy. Early detection of aneurysmal changes can be life-saving, and this study moves us closer to that goal by validating ultrasound’s utility in a controlled experimental context.

On the therapeutic front, one paper in the issue highlights an emerging pharmacological strategy targeting vascular remodeling and dysfunction in a severe cardiovascular condition. Sánchez-Gloria et al. investigated the compound allicin—a bioactive molecule derived from garlic—as a potential treatment for pulmonary arterial hypertension (PAH) [19]. PAH is a progressive vascular disease characterized by high pulmonary blood pressure, vascular remodeling in the lungs, and right heart failure [20]. The authors hypothesized that allicin’s known antioxidant and antihypertensive properties might ameliorate the pathological changes in PAH. In an experimental rat model of PAH (induced by monocrotaline toxin), allicin administration produced remarkably positive outcomes. Treated rats showed improved body weight and survival, indicating an overall mitigation of disease severity. Allicin prevented the typical vascular remodeling seen in PAH: it reduced or normalized the medial wall thickening of pulmonary arteries and averted right ventricular hypertrophy, both of which are hallmark indicators of disease. Mechanistically, the therapy modulated several key molecular pathways. Allicin-treated animals maintained normal levels of angiotensin II and its receptors in lung tissue, pointing to a beneficial effect on the renin–angiotensin system. It also enhanced the bioavailability of nitric oxide (NO) and related signaling molecules (such as cyclic GMP and the cofactor tetrahydrobiopterin) in the pulmonary circulation, which would promote vasodilation and endothelial function. Furthermore, allicin boosted the antioxidant defense (e.g., upregulating Nrf2 expression) and prevented hypoxia-driven proliferative signaling by suppressing HIF-1α and VEGF overexpression in the lungs. Collectively, these effects translated into attenuated pulmonary vascular remodeling and preservation of heart function. By the end of the study, the authors conclude that allicin’s global multimodal effects—reducing oxidative stress, modulating vasoactive peptides, and improving endothelial function—make it a promising adjunct or alternative therapy for PAH. They caution that human trials are needed, but the data suggest allicin could be considered when current treatments are insufficient or when a combination approach is required to address multiple pathogenic mechanisms. The implications of this work are significant: it exemplifies how targeting molecular pathways (oxidative stress, nitric oxide signaling, growth factor responses) can reverse or halt severe vascular disease in vivo. Allicin, or drugs with similar pleiotropic effects, might one day complement existing PAH therapies and could even inspire treatments for other cardiovascular diseases marked by inflammation and remodeling, such as systemic hypertension or aneurysms.

## 3. Conclusions and Acknowledgments

In summary, the collection of papers in the “Molecular Mechanisms, Pathogenesis, and Novel Therapeutic Strategies in Cardiovascular Disease” special issue offers a comprehensive look at the state-of-the-art in aneurysm research and related vascular conditions. From unraveling the molecular pathology of aneurysms—including how bacteria, genes, and immune cells conspire to weaken the vessel wall—to developing novel strategies for diagnosis and treatment, the contributions herein push the frontier of our understanding. Equally important, they highlight the translational potential of this knowledge: the prospect of new diagnostics and therapies that can improve patient outcomes in the future. While significant challenges remain (for example, translating these findings into clinical trials, and ensuring safety and efficacy in humans), the advances documented in this issue lay a strong foundation for the next steps in research and innovation.

On behalf of the Guest Editor and the journal, we extend our sincere gratitude to all the authors who contributed their cutting-edge research to this special issue. Their diligent work and insightful findings have made this collection possible. We also thank the many reviewers and editors who provided constructive feedback, ensuring the high quality and rigor of the published papers. I and the editorial team are confident that these articles will stimulate further inquiry and collaboration in the field. We encourage researchers to build upon these findings—to explore unanswered questions about aneurysm initiation and progression, to validate these concepts in clinical settings, and to ultimately develop effective diagnostic tools and therapies for cardiovascular diseases. By continuing to probe the molecular mechanisms and test novel strategies, the scientific community moves ever closer to mitigating the burden of aneurysms and related cardiovascular disorders. The hope is that what has been learned and shared in this special issue will inspire ongoing innovation and, in time, translate into tangible health benefits for patients at risk of these devastating diseases.
